# Cost-consequence analysis of influenza vaccination among the staff of a large teaching hospital in Rome, Italy: A pilot study

**DOI:** 10.1371/journal.pone.0225326

**Published:** 2019-11-14

**Authors:** Vittoria Colamesta, Andrea Tamburrano, Andrea Barbara, Andrea Gentili, Daniele Ignazio La Milia, Filippo Berloco, Americo Cicchetti, Daniele Piacentini, Roberta Galluzzi, Sergio Riccardo Mastrodonato, Andrea Cambieri, Walter Ricciardi, Patrizia Laurenti

**Affiliations:** 1 Università Cattolica del Sacro Cuore, Section of Hygiene - Institute of Public Health, Roma, Italia; 2 Local Health Unit ASL RM1, UOC Direzione Sanitaria S. Spirito e Nuovo Regina Margherita, Roma, Italia; 3 Fondazione Policlinico Universitario A. Gemelli IRCCS, Hospital Health Management, Roma, Italia; 4 Università Cattolica del Sacro Cuore, Postgraduate School of Health Economics and Management (ALTEMS), Roma, Italia; 5 Fondazione Policlinico Universitario A. Gemelli IRCCS, Human Resources Management, Roma, Italia; 6 Fondazione Policlinico Universitario A. Gemelli IRCCS, Department of Woman and Child Health and Public Health, Roma, Italia; National Center for Global Health and Medicine, JAPAN

## Abstract

Flu vaccination, as well as being effective to prevent seasonal influenza, decreases staff illness and absenteeism and reduces costs resulting from loss of productivity. Despite the effectiveness of flu vaccination, the seasonal coverage among healthcare workers is usually low. The aim of this retrospective observational study was to analyze the vaccination coverage rate among all employees (healthcare workers and administrative staff) of a large teaching hospital in Rome during the 2017–2018 influenza season, to perform a cost-consequence analysis of influenza vaccination (by evaluating the absenteeism due to illness in the epidemic period), and to assess the impact of vaccination in terms of both costs and sick days. The flu vaccination coverage rate was 9.8% among 4631 healthcare workers and 852 administrative employees. The human capital approach estimated a loss of productivity equal to 297.06 € for each vaccinated worker and 517.22 € for each unvaccinated worker (cost-outcome ratio: 120.07 €/sick day). Applying the friction cost method, a loss of productivity equal to 237.65 € for each vaccinated worker and 413.78 € for each unvaccinated worker (cost-outcome ratio: 104.19 €/sick day) was found. These results confirm the benefits of the flu vaccination for the society and the company. This allowed the management to grant one hour of permission to the flu-vaccinated workers in the following annual vaccination campaign (2018–2019).

## Introduction

Influenza is a highly contagious but preventable acute respiratory illness that affects approximately 10% of general population every year and that is associated with increased morbidity and mortality in high risk groups [1]. The World Health Organization (WHO) estimated up to 5 million cases of severe illness and between 250,000 and 500,000 deaths occur each year worldwide as result of seasonal influenza epidemics [2].

Annual vaccination represents the most effective way to prevent the infection [3, 4]. The increase of seasonal coverage represents an important concern for national and international organizations. A European qualitative study carried out by European Centre for Disease Prevention and Control (ECDC), through 65 semi-structured interviews with vaccine providers in Croatia, France, Greece, and Romania, reported that the major determinants of vaccination coverage are country and context-specific. Strategies to improve confidence in vaccines should therefore involve political, social, cultural and economic framework [5]. In this perspective, the WHO recommends flu vaccination both in groups considered to be at high risk of exposure to the influenza virus and in those at risk of developing severe disease, including healthcare workers (HCWs), pregnant women, children aged between 6 months to 5 years, elderly individuals (aged more than 65 years) and individuals with underlying health conditions such as HIV/AIDS, asthma, and chronic heart or lung diseases [6]. Even in Italy, following the WHO advice, the National Vaccine Prevention Plan (*Piano Nazionale Prevenzione Vaccinale*–PNPV) 2017–2019 strongly recommends the flu vaccine to elderly, people suffering from specific conditions of risk (diabetes, cardiovascular and respiratory diseases, cancer, etc.) and HCWs with the goal to achieve the 75% flu vaccination coverage, as minimum target, and 95% as optimal one [7].

Despite the effectiveness of flu vaccination and the strong recommendations provided by WHO and the Italian PNPV, the seasonal flu vaccination coverage among HCWs in Europe and in Italy is usually low, seldom exceeding 30%. In most European countries, during the influenza season 2016–2017, flu vaccination coverage was less than 40%, with a median of 30.2% (range: 15.6%-64.6%) [8]. In Italy, instead, looking at two systematic reviews, it is possible to estimate the influenza vaccination coverage rate among HCWs in Italy between 13% (for nurses) and 23% (for physicians) [9, 10]. Moreover, significant differences are achieved among seasons, regions, hospitals, operative units and categories of HCWs too. For example, the vaccination coverage rate among HCWs in “P. Giaccone” teaching hospital located in Palermo varied from 14.7% in 2005–2006 to 8.2% in 2007–2008 and 3.1% in the 2011–2012 influenza season [11]. In the “Ospedale Maggiore Policlinico, Mangiagalli e Regina Elena” hospital of Milan [12], during the 2005–2006 season, flu vaccination coverage rate ranged from 17.6% in an emergency unit to 24.3% in a surgery unit. In a regional tertiary adult acute-care reference center with a 1,300-bed capacity in Genoa, during the influenza season 2013–2014 a flu vaccination coverage rate of 30% among physicians, 11% among nurses and 9% among other clinical personnel was reported [13]. In our teaching hospital in Rome, after the original experience of "Forum Theater" to promote positive vaccination attitudes [14], starting from the 2015–2016 flu season the Hospital Health Management organized specific sessions of on-site vaccination. It consists in providing free of charge vaccination directly to hospital wards, in addition to vaccination offered during the daily activity by the hospital’s Preventive Medicine Service, according to a specific time schedule. This strategy resulted effective in achieving higher flu vaccination coverage among HCWs [15] and resident doctors [16], even though it was less than 20% overall.

A recent Italian report evaluated the economic and fiscal impact of influenza, pneumococcal and herpes-zoster vaccines in Italy, even though it was not focused on HCWs. In the cost-benefit analysis carried out in the article, the authors found that investing in the flu vaccination, the benefits *per capita* amount to 1.8 times the value of investment in terms of fiscal impact and 11.1 times in terms of productivity loss [17]. Another Italian study, focused on HCWs, shows an increase in sick leave during the flu epidemic period among all HCWs categories (medical doctors, technical executives, nurses and allied health professionals, other executives, non-medical support staff, administrative staff). In addition, vaccinated HCWs showed a reduced absenteeism compared to unvaccinated ones (1.45 vs 2.09 days/person) [18]. In the previous mentioned teaching hospital in Palermo, during the 2007–2008 season the vaccination rate was 8.2% and the absenteeism due to Influenza-Like Illness (ILI) was significantly higher in the unvaccinated HCWs, determining the spreads of the influenza virus and provoking an avoidable cost for the hospital and for the community [19]. In another Italian teaching hospital located in Modena the prevalence of ILI in the unvaccinated group was significantly increased compared to vaccinated subject (24% vs 15% respectively; p<0.001). Working days lost for ILI were 516 in the unvaccinated group versus 315 reported in the vaccinated group. Economic impact evaluation showed a cost of € 35,736.88 in the vaccinated and of € 57,759.52 in the unvaccinated. The resulting indirect benefit (IB) was € 21,078.64, the direct cost (DC) and indirect cost (IC) for vaccination were €2,463.29 and € 2,172.53 respectively and the overall cost-benefit ratio IB/(DC+IC) was 4.5 [20].

The aim of the present study was to analyze the vaccination coverage rate among all employees (healthcare workers and administrative staff) in a large teaching hospital in Rome during the 2017–2018 influenza season, to perform a cost-consequence analysis of influenza vaccination through an analysis of absenteeism due to illness in the epidemic period, and to compare the impact of vaccination in terms of both costs and sick days.

## Materials and methods

### Ethics statement

This study is compliant with the Local Ethical Committee Standards of the Fondazione Policlinico Universitario Agostino Gemelli IRCCS. It was approved and registered with number 2018-04/23/2018 and was carried out in accordance with the Helsinki Declaration and EU Regulation 2016/679 (GDPR) concerning the processing of personal data. For this type of study, Ethical Committee did not foresee the need for participant consent.

### Setting

A retrospective observational study was carried out in our teaching hospital in Rome, analyzing data from the 2017–2018 influenza season. This is an acute-care reference center with a 1,500-bed capacity and almost 5,500 workers employed.

### Flu vaccination strategy, coverage and absenteeism due to influenza

Vaccination was offered to all staff as a part of the hospital prevention program; it was administered by the hospital’s Preventive Medicine Service and performed with quadrivalent flu vaccine *Fluarix*^®^ *Tetra* (split, inactivated, non-adjuvanted). During the 2017–2018 influenza vaccination campaign, training sessions on flu vaccination have been organized according to Academic Detailing methodology [21]. The purpose was to deliver tailored training and technical assistance to healthcare providers to help them using best practices on influenza prevention and control [21]. Vaccinated and unvaccinated hospital workers (health workers and administrative staff) and the absenteeism (number of working days lost for illness—namely, sick days—during the epidemic flu period) in both groups have been counted and compared. Based on the fact that exposure to other possible causes of illness (including circulating infectious diseases other than influenza, for example other respiratory viruses) was the same for both groups, it was assumed that the difference in absences was only due to influenza. Working days were considered lost for illness when the mandatory medical certificate for sick leave was sent by the worker to the hospital health management (his own employer), as requested by the Italian law. The epidemic period was considered in accordance to Influnet [22], an Italian nationwide sentinel surveillance network of influenza epidemiology that collects clinical and virologic information about influenza from week 42 to week 17 of the following year.

Descriptive statistic was performed analyzing mean and Standard Deviation (SD) of quantitative variables and frequencies and percentages of qualitative ones. Workers job categories have been grouped into physicians, nurses, administrative staff, other graduated and ungraduated health professionals. Moreover, t-Student test was used to assess differences in absenteeism due to illness between vaccinated and unvaccinated workers, considering all employees and then stratifying by job categories. The level of statistical significance was set at p<0.05.

### Cost-consequence analysis

To compare the impact of flu vaccination among hospital workers, in terms of both costs and sick days, a cost-consequence approach was used [23]. Through this methodology the impact of the vaccination on short-time (epidemic period) resource use and costs (productivity losses) and health outcomes (sick days) was estimated and presented in a tabular format. The analysis was performed through a 6-months horizon, no discount rate was applied, adjustment for a common base year was not necessary.

Direct medical costs referred to the acquisition costs of the vaccine (ex-factory price of 11.08 €) [24, 25] and the administration costs of the vaccination (set to 6.16 €) [24, 26, 27].

Indirect costs referred to costs falling outside the healthcare sector and concerned productivity lost (workdays/hours lost) due to influenza. Daily/hour wage was set in accordance with the national collective agreement [28].

The loss of productivity (linked to sick leave) for the society and for the company was estimated by using the human capital approach and the friction cost method, respectively.

The human capital approach estimates the value of lost production due to temporary absence from work for sick leave. The measure of income can be obtained through the sample estimate of individual patient income or, alternatively, through the remuneration received by workers classified by sector of activity and professional categories, detectable from surveys conducted by national institutions responsible for the management of tax revenue [29].

The friction cost method assumes that the value of lost production associated with a disease depends on the period necessary to retrieve the original level of production (during the absences the work can be done by colleagues—friction period). It has been estimated that the productivity reduction deriving from one day of absence from work is equal to about 80% of the sick worker's daily income [30]. Therefore, this value of productivity loss was applied.

Direct costs were considered only for the human capital approach, because of for the company perspective (friction cost method) the vaccine was provided free of charge by the Italian NHS and administered by the hospital’s Preventive Medicine Service without additional usage of resources and working hours.

Finally, the cost-outcome ratio was calculated by dividing the difference in total costs (vaccination costs, where appropriate, and loss of productivity) *per capita* among vaccinated and unvaccinated workers by the difference between the mean of absenteeism among vaccinated and unvaccinated workers.

## Results

### Flu vaccination coverage and absenteeism due to influenza

The teaching hospital counted 5,483 hospital workers employed and all of them were included in the study. During the flu vaccination season 2017–2018, 538 of them (9.8%) have been vaccinated. In Table 1 the main sample characteristics are reported, according to flu vaccination status. Considering the job categories, physicians had the highest immunization coverage (21.9% of doctors have been vaccinated, compared to 8.5% of nurses, 10.2% of graduated health professionals and less than 5% of other categories).

**Table 1:** Main characteristics of hospital workers. Row percentages are reported.

Variables		Vaccinated N = 538 (9.8%)	Unvaccinated N = 4,945 (90.2%)	Total N = 5,483
Age *(mean ± SD)*		48.92 ± 10.8	45.55 ± 10.7	45.88 ± 10.7
Sex	*Male*	264 (11.9%)	1,955 (88.1%)	2,219
	*Female*	274 (8.4%)	2,990 (91.6%)	3,264
Job categories	*Physicians*	233 (21.9%)	831 (78.1%)	1,064
	*Nurses*	177 (8.5%)	1,898 (91.5%)	2,075
	*Graduated health professionals*	59 (10.2%)	519 (89.8%)	578
	*Ungraduated health professionals*	45 (4.9%)	869 (95.1%)	914
	*Administrative staff*	24 (2.8%)	828 (97.2%)	852

Regarding the absenteeism during the epidemic flu period, a mean of 1.6 ± 6.0 days of absence from work for sick leave was observed among vaccinated and a mean of 3.3 ± 10.9 days among unvaccinated hospital workers (mean difference = 1.7; p<0.001) (Table 2). Moreover, nursing absenteeism was 2.8 ± 7.1 days among vaccinated and 4.2 ± 12.3 days among unvaccinated employee, while physicians’ absences were close to 0 for both groups in the epidemic period.

**Table 2:** Absenteeism (number of working days lost) during the epidemic flu period compared between vaccinated and unvaccinated hospital workers.

Job categories	Absenteeism		
	Vaccinated (mean ± SD)	Unvaccinated (mean ± SD)	p-value
*Physicians*	0.0 ± 0.0	0.0 ± 0.1	1.000
*Nurses*	2.8 ± 7.1	4.2 ± 12.3	0.136
*Graduated health professionals*	3.1 ± 6.8	2.5 ± 8.4	0.597
*Ungraduated health professionals*	2.0 ± 4.1	4.0 ± 11.8	0.257
*Administrative staff*	4.8 ± 15.9	4.2 ± 12.8	0.822
**Total**	**1.6 ± 6.0**	**3.3 ± 10.9**	**<0.001**

### Cost-consequence analysis

In Table 3 the results of the cost-consequence analysis are described.

**Table 3:** Results of the cost-consequence analysis (loss of productivity and cost-outcome ratio) during the epidemic flu period.

		Human capital approach	Friction cost method
*Loss of productivity (per capita)*	Vaccinated hospital workers	297.06 €	237.65 €
	Unvaccinated hospital workers	517.22 €	413.78 €
Loss of productivity (all employees*)	Vaccinated hospital workers	1,628,765 €	1,303,012 €
	Unvaccinated hospital workers	2,835,916 €	2,268,733 €
**Cost-outcome ratio**		**120.07 €/sick day**	104.19 €/sick day**

* Assuming everyone vs no one is vaccinated.

** Direct costs not included.

The human capital approach estimated, during the flu epidemic period, a loss of productivity equal to 297.06 € for each vaccinated and 517.22 € for each unvaccinated hospital worker (cost-outcome ratio = 120.07 €/sick day). Referring to all employees, a total loss of productivity of 1,628,765 € avoided for vaccinated and 2,835,916 € avoided for unvaccinated hospital workers has been found.

The friction cost method estimated, during the epidemic flu period, a loss of productivity equal to 237.65 € for each vaccinated and 413.78 € for each unvaccinated hospital worker (cost-outcome ratio = 104.19 €/sick day). Referring to all employees, a total loss of productivity of 1,303,012 € avoided for vaccinated and 2,268,733 € avoided for unvaccinated hospital workers has been found.

## Discussion

The present study highlights the economic value of flu vaccination among hospital workers (healthcare workers and administrative staff) in a large Italian teaching hospital. The cost-consequence analysis suggests an economic advantage for the society and for the company (as well as in terms of health) to provide flu vaccination to hospital workers.

In the performed pilot study, a flu vaccination coverage in hospital workers of almost 10% was found, with substantial differences among the identified job categories. A statistically significant difference in absenteeism between vaccinated and unvaccinated hospital workers was also observed. According to our knowledge, a higher excess of absenteeism rate among unvaccinated compared to vaccinated healthcare workers during the epidemic period (2.09 vs 1.45 days/person) was found also by Gianino et al. [18] in a recent Italian retrospective observational study. Similar results were detected even by the previously mentioned Italian study conducted in Palermo and Modena [11, 20].

### Limitations

This study did not analyze differences in absenteeism rate between the epidemic and the non-epidemic period, focusing only on the first one. This could be a limit because several studies reported an absenteeism that range between the 35% and 2-fold higher during epidemic period compared to the rest of the year [31, 32], despite other studies highlighted few differences [33]. For example, Gianino et al. [18] founded difference in absenteeism between vaccinated and unvaccinated healthcare workers also during the non-epidemic period and explained this discrepancy assuming that vaccines also protect employees from illness during non-epidemic period.

In addition, this study was based on a prevention practice conducted “on the field”, offering vaccination to all hospital workers without randomization or sample size calculation. Despite this, the hospital prevention program guaranteed free access to vaccination and allowed to have a much larger population in both groups.

Moreover, in this pilot study it was assumed that the difference in absences between groups during the epidemic period was mostly due to the flu vaccination intervention. Indeed, as mentioned above, we assumed that the exposition to other possible causes of illness during the epidemic period (including typical circulating infectious diseases of the wintertime, not vaccine-preventable, i.e. other respiratory viruses) was the same, considering the high number of people in the two groups too. Besides, the absenteeism has not been stratified by cause of illness because of the privacy obligations that do not allow the employer to know the diagnosis of sick leave for which the hospital workers employed were absent.

Furthermore, the study design is based on a static model. Despite this does not consider the indirect effect of vaccination on the unvaccinated individuals (a worker is more protected from infection if a colleague is vaccinated—herd immunity effect), as in dynamic model [34, 35], the low vaccination coverage rate among all job categories make this effect almost negligible [36, 37]. This study design limitation bounds the possibility of extrapolating the results by assuming the condition in which everyone is vaccinated because of in this case the indirect effect is no longer negligible.

Finally, although cost-consequence analysis is sometimes considered less rigorous than other economic evaluations, it is at the same time more versatile and practical being able to offer clear and simple information thus representing a valid framework for the appraisal of flu vaccination in the Italian context.

## Conclusions

Despite the inherent limitations discussed, the current work represents a pilot study performing an economic evaluation of flu vaccination among hospital workers in a large teaching hospital in Italy. The above mentioned results confirm the benefits of flu vaccination for the society and for the company. These findings also oriented the hospital health management to encourage vaccination by giving an additional hour of leave to the flu-vaccinated workers in the following annual vaccination campaign (2018–2019).

## Supporting information

S1 File Data set (extraction from human resources and preventive medicine service information systems). (XLSX)
